# Evaluation of the Cardiovascular and Serotonergic Modulatory Effects of Ondansetron in Healthy Dogs Under Anesthesia

**DOI:** 10.3390/vetsci13020119

**Published:** 2026-01-27

**Authors:** Giovanna Lucrezia Costa, Nicola Maria Iannelli, Fabio Bruno, Stefania Turco, Annamaria Passantino, Caroline Munhoz, Patrizia Licata, Michela Pugliese

**Affiliations:** 1 Department of Veterinary Sciences, Univerity of Messina, 98168 Messina, Italy; glcosta@unime.it (G.L.C.); nicolamaria.iannelli@unime.it (N.M.I.); plicata@unime.it (P.L.); michela.pugliese@unime.it (M.P.); 2 Provincial Health Company, 93100 Caltanissetta, Italy; drstefaniaturco@gmail.com; 3 Veterinary Practitioner, Belo Horizonte 30110, Brazil

**Keywords:** ondansetron, atropine, anesthesia, autonomic modulation, NT-proBNP, dogs

## Abstract

Maintaining cardiovascular stability during anesthesia is essential, yet the routine use of atropine to prevent vagally induced low heart rate may impose additional stress on the heart. This randomized, controlled, observer-blinded, clinical study aimed to evaluate whether ondansetron, a selective 5-HT_3_ receptor antagonist, could serve as an alternative anesthetic adjuvant to modulate autonomic activity while maintaining cardiovascular stability in dogs. A total of 66 female dogs, with a mean age of 1.5 years and a mean weight of 16–18 kg ASA I, undergoing elective surgery were assigned to three study groups to receive atropine, ondansetron, or no autonomic-modulating drug. Heart rate, arterial pressure, respiratory rate, and NT-proBNP were recorded before, during, and after anesthesia. Dogs treated with ondansetron maintained stable cardiovascular values throughout the procedure, with no episodes of low heart rate or excessive increases in heart rate. In contrast, atropine induced marked and sustained elevation in heart rate and higher arterial pressures. Concentrations of the cardiac biomarker NT-proBNP increased significantly 48 h after surgery in the atropine group but remained unchanged in the ondansetron group, indicating the absence of additional myocardial stress. These findings suggest that ondansetron may help preserve autonomic balance during anesthesia while minimizing myocardial stress. Ondansetron could represent a useful component of multimodal anesthetic protocols, particularly in dogs in which excessive cardiac stimulation should be avoided.

## Simple Summary

Maintaining autonomic and cardiovascular stability during anesthesia is essential for the safety of dogs. Atropine is commonly administered to prevent vagally mediated bradycardia; however, it may induce excessive sympathetic stimulation, resulting in marked tachycardia and increased cardiac workload. This study evaluated whether ondansetron, a serotonin 5-hydroxytryptamine type 3 (5-HT3) receptor antagonist primarily used as an antiemetic, could serve as a safer anesthetic adjuvant for autonomic modulation during anesthesia. Healthy dogs undergoing elective surgery received atropine, ondansetron, or no autonomic-modulating drug. Heart rate, blood pressure, respiratory rate, and a cardiac biomarker of myocardial stress, N-terminal pro–B-type natriuretic peptide (NT-proBNP), were assessed before, during, and after anesthesia. Dogs treated with ondansetron maintained stable cardiovascular parameters without developing bradycardia or excessive tachycardia, whereas atropine administration was associated with a sustained increase in heart rate and a postoperative rise in NT-proBNP concentrations. These findings suggest that ondansetron may help preserve autonomic balance during anesthesia while minimizing myocardial stress. Ondansetron could represent a useful component of multimodal anesthetic protocols, particularly in dogs in which excessive cardiac stimulation should be avoided.

## 1. Introduction

Recent pharmacological developments in the field of antiserotoninergic drugs have led to the introduction of 5-HT3 antagonists, including ondansetron, which is employed in the management of chemoradiation-induced vomiting [1]. In addition, 5-HT3 antagonists have been investigated as a potential component of combination therapy, particularly in conjunction with cytotoxic drugs, which have both antitumor and immunosuppressive properties [2].

At the central level, the antiemetic mechanism of ondansetron is mediated by suppression of 5-HT3 receptor activity in the chemoreceptor trigger zone (CTZ), also referred to as the area postrema. This structure is anatomically located on the dorsal surface of the medulla oblongata at the base of the fourth ventricle [3]. The CTZ detects circulating emetogenic agents and transmits this information directly to the vomiting center, which initiates the emetic reflex [3].

In addition to its action on the CTZ, ondansetron appears to exert effects on other central nervous system structures, including the nucleus of the solitary tract, amygdala, and dorsal raphe nucleus, with probable inhibition of dopamine release in the nucleus accumbens [3,4]. These regions are involved in the integration of visceral afferent inputs and autonomic cardiovascular regulation.

At the peripheral level, ondansetron counteracts the effects induced by high levels of 5-HT released by enterochromaffin cells in the intestinal mucosa when tissue damage occurs, such as during exposure to cytotoxic drugs [5]. Under these conditions, serotonin stimulates 5-HT3 receptors located on the synaptic terminals of the vagus nerve, leading to strong activation of afferent pathways and transmission of the emetogenic stimulus to higher centers [6].

Ondansetron exerts its pharmacological action by selectively blocking depolarisation of both vagal afferent fibers and myenteric neurons, resulting in attenuation of nociceptive and reflex responses mediated by 5-HT3 receptors [6]. Notably, ondansetron exhibits a selectivity ratio of approximately 1000:1 for the 5-HT3 receptor, while showing weaker affinity for 5-HT1B, 5-HT1C, 5-HT4, α1-adrenergic, and μ-type opioid receptors [6,7]. The antagonistic activity of ondansetron on 5-HT3 receptors has been demonstrated both in vitro in rat neuronal cells and in vivo in anesthetized rats and cats [6].

Ondansetron may be administered orally, together with food and antacids, without altering the absorption of the active substance, or via slow intravenous infusion [1,8]. Oral administration results in a bioavailability of approximately 60%, largely due to first-pass metabolism and plasma protein binding, with about 75% of the drug remaining bound [1,8]. Peak plasma concentrations occur approximately 1.5 h after oral administration, while cerebrospinal fluid concentrations are reduced by less than 15% compared to plasma levels [8,9,10].

The drug undergoes phase I hydroxylation mediated by cytochromes P450 1A2, 2D6, and 3A4, followed by phase II conjugation with glucuronic acid and sulfates in the liver [10,11]. Inactive metabolites are eliminated via urine and feces, resulting in a maximum half-life of approximately 4 h and a clearance of about 541 mL/min [12].

Although the high cost of antiemetics limits their routine use in veterinary practice, ondansetron is prescribed in dogs and cats for the treatment of vomiting associated with chemotherapy, radiotherapy, gastrointestinal viral diseases, or in cases where conventional treatments are ineffective [13].

Ondansetron inhibits serotonergic and vagal activity, thereby preventing vomiting, but does not exhibit dopaminergic or muscarinic receptor agonist activity [13]. Dopamine plays a key role in regulating behavior, voluntary movement, cognition, motivation, sleep, and mood, and activates the sympathetic nervous system, leading to increased heart rate and arterial blood pressure [13]. Muscarinic (cholinergic) actions correspond to those of acetylcholine released from parasympathetic postganglionic nerve terminals, with the notable exception that acetylcholine induces generalized vasodilation, even in vascular beds not directly innervated by the parasympathetic system [13].

Increasing evidence suggests that ondansetron, through selective antagonism of 5-HT3 receptors, is capable of modulating autonomic nervous system activity, particularly by influencing vagal afferent pathways [2]. This pharmacological profile is especially relevant in anesthetic practice, where preservation of autonomic balance is essential to maintain cardiovascular stability.

The stability of the autonomic nervous system is crucial during anesthesia and surgical stress, as excessive vagal or sympathetic activation can lead to significant cardiovascular alterations, including bradycardia, hypotension, or arrhythmias [14]. Surgical stress induces a complex autonomic response, characterized by increased sympathetic tone, catecholamine release, and modulation of vagal activity, which may exacerbate myocardial workload and contribute to perioperative cardiovascular complications [15,16,17,18,19]. Pharmacological modulation of this response—for instance, by using drugs that attenuate excessive autonomic activation—can help maintain cardiovascular stability and reduce myocardial stress. Ondansetron, through selective 5-HT3 receptor antagonism, has been shown to influence vagal afferent pathways and modulate autonomic balance, suggesting a potential role in mitigating vagally mediated bradycardia during anesthesia [2,16].

ProBNP, specifically NT-proBNP, is a circulating biomarker released by cardiac myocytes in response to myocardial stress or damage. In dogs, postoperative NT-proBNP concentrations may reflect cardiac strain associated with surgical stress, anesthesia, or pain and can serve as both diagnostic and prognostic indicators of perioperative cardiovascular complications [13,20,21,22,23,24].

On the basis of these considerations, the objective of the present study was to evaluate whether ondansetron can be used as an alternative anesthetic adjuvant to atropine for the prevention of vagally mediated bradycardia in dogs, while maintaining cardiovascular stability and minimizing myocardial stress. Specifically, the effects of ondansetron on heart rate, respiratory rate, arterial blood pressure, and NT-proBNP concentrations were compared with those observed following atropine administration and with a control protocol lacking autonomic-modulating drugs.

We hypothesized that ondansetron would preserve autonomic balance during anesthesia, preventing bradycardia without inducing excessive sympathetic activation, and would not be associated with postoperative increases in NT-proBNP concentrations. By addressing the need for anesthetic strategies that support autonomic stability while reducing cardiac workload, this study aims to provide clinically relevant evidence for the incorporation of ondansetron into multimodal anesthetic protocols in dogs.

## 2. Materials and Methods

### 2.1. Ethical Approval and Study Population

The study protocol was reviewed and approved by the Ethics Committee of the University of Messina (Protocol No. 11/2024). Furthermore, we confirm that the study adhered to the ARRIVE guidelines for reporting animal research, ensuring rigorous and transparent reporting of experimental design, animal welfare, and ethical considerations. Only dogs classified as ASA I (American Society of Anesthesiologists physical status I) were included thorough physical examination, complete blood count (CBC), serum biochemistry, and evaluation of general health status. Subjects not belonging to this class were excluded from the study. Written informed consent was obtained from all owners prior to enrolment.

A total of 66 female dogs were enrolled in the study, with an age of 1.5 ± 0.5 years, a mean weight of 17 ± 1.41 kg and a BCS of 4–5/9. Age, body weight, and BCS did not differ significantly among groups (*p* > 0.05), and all underwent elective ovariectomy via laparotomy. Preoperative assessment included a complete physical examination and routine investigations, including CBC, serum biochemistry, NT-proBNP (N-terminal pro–B-type natriuretic peptide) assay, and echocardiographic evaluation with two-dimensional, M-mode, and Doppler examination, performed by a board-certified cardiologist to exclude congenital or acquired cardiac disease. Serum biochemistry included AST (aspartate aminotransferase), ALT (alanine aminotransferase), total protein, albumin, glucose, and BUN (blood urea nitrogen). Dogs with cardiac disease, hematological abnormalities, or any systemic condition incompatible with ASA I classification were excluded.

### 2.2. Study Design, Randomization, and Group Allocation

This study was designed as a prospective, randomized, observer-blinded clinical trial. A total of 66 dogs were enrolled and randomly allocated by lottery to one of three treatment groups (n = 22 per group). The investigator responsible for intraoperative monitoring and postoperative assessment was blinded to group assignment.

Dogs received one of the following premedication protocols:

**Group C (Control):** Methadone 1% (0.3 mg/kg IV; Semfortan^®^, Dechra, The Netherlands) combined with diazepam (0.25 mg/kg IV; Ziapam^®^, Domes Pharma SC, Pont-du-Château, France), administered in separate syringes [25].

**Group O (Ondansetron):** Ondansetron 0.02% (0.5 mg/kg IV; Ondansetron Accord^®^, Accord Healthcare Italia S.r.l., Milano, Italy) in combination with methadone (0.3 mg/kg IV) and diazepam (0.25 mg/kg IV) [26].

**Group A (Atropine):** Atropine sulfate (0.02 mg/kg IV; Atropine Sulfate^®^ 0.1%, A.T.I., Ozzano dell’Emilia, Italy) combined with methadone (0.3 mg/kg IV) and diazepam (0.25 mg/kg IV) [27].

### 2.3. Anesthesia Protocol

All dogs were premedicated according to group allocation. Premedication was administered approximately 20 min before anesthetic induction.

Anesthesia was induced with propofol (Proposure 1%, Merial, Milano, Italy) administered intravenously to effect, at a recorded mean dose of 4–6 mg/kg, until loss of jaw tone and abolition of the palpebral reflex were achieved [27].

Orotracheal intubation was then performed under direct laryngoscopic visualization using cuffed endotracheal tubes of appropriate internal diameter (6.0–8.5 mm), selected according to the dog’s body size and airway anatomy. Correct tube placement was confirmed by visualization of the tube passing through the vocal cords and by the presence of end-tidal CO_2_.

Anesthesia was maintained with isoflurane (Isoflo, Esteve, Milano, Italy) in 100% oxygen delivered via a non-rebreathing system (MATRX VMS, Alcyon, Cherasco, Italy) [28]. All dogs were allowed to breathe spontaneously throughout anesthesia. The vaporizer setting was adjusted to maintain an adequate depth of anesthesia, corresponding to approximately 1.2–1.5 times the minimum alveolar concentration (MAC) of isoflurane in dogs. Depth of anesthesia was assessed throughout the procedure based on clinical parameters, including heart rate, respiratory rate, arterial blood pressure, jaw tone, palpebral reflex, and response to surgical stimulation.

All dogs were allowed to breathe spontaneously, and mechanical ventilation was not required. Intraoperative analgesia was provided by a lidocaine 2 mg/kg 2% (Lidocaine Esteve) splash block applied to each ovarian pedicle [25].

Rescue analgesia was administered with fentanyl (Fentadon, Dechra, Bladel, The Netherlands) at a dose of 2 μg/kg IV if signs of inadequate analgesia or sympathetic stimulation were observed [29].

### 2.4. Hemodynamic Monitoring and Intraoperative Nociception Assessment

Hemodynamic monitoring was performed using a multiparameter monitor (EDAN Instruments, Naples, Italy) at the following time points:

T1: Baseline, after acclimation;

T2: 5 min after premedication;

T3: Induction of anesthesia;

T4: Skin incision;

T5: Muscle incision;

T6: Ovariectomy (traction and removal of the second ovary);

T7: Skin closure.

Recorded parameters included heart rate (HR), respiratory rate (fR), systolic arterial pressure (SAP), diastolic arterial pressure (DAP), and mean arterial pressure (MAP), all continuously monitored using a multiparametric anesthetic monitor (EDAN Instruments, Naples, Italy). Heart rate was measured by electrocardiography (ECG), respiratory rate was derived from capnography, and arterial blood pressure was measured noninvasively using an oscillometric technique. An appropriately sized cuff (width approximately 40% of limb circumference) was placed on a thoracic limb in all dogs. End-tidal carbon dioxide (EtCO_2_) was measured by sidestream capnography via the endotracheal tube, while arterial oxygen saturation (SpO_2_) was assessed using pulse oximetry with the probe positioned on the tongue or lip. Rectal temperature was monitored using a digital rectal thermometer.

To, EtCO_2_ and SpO_2_ were measured noninvasively using the anesthetic monitoring system.

Intraoperative nociception was assessed using a cumulative pain scale based on percentage changes in relative HR, fR, and SAP to stabilized baseline values under general anesthesia. A total score > 10, corresponding to a ≥20% increase in these parameters, indicated severe pain and triggered rescue fentanyl administration [30].

All surgical steps and corresponding time points (T3–T7) were standardized and performed in the same chronological sequence for all dogs, with comparable durations.

All ovariectomies were performed by the same experienced surgeon, minimizing inter-operator variability.

### 2.5. Postoperative Analgesia and Monitoring

Postoperatively, all dogs received meloxicam (0.1 mg/kg orally every 24 h; Metacam, Boehringer Ingelheim, Milano, Italy) for 24 h. Pain assessment was performed every 4 h for 24 h using the Colorado pain scale. A score ≥ 2 (moderate pain) was the cut-off for rescue analgesia, administered as methadone (0.3 mg/kg intramuscularly). Dogs were hospitalized for at least 24 h and discharged after 5 days following a clinical check-up.

### 2.6. Blood Sampling and Biochemical Analysis

Blood samples were collected at baseline (T0) and 48 h post-surgery (T8). A total of 5 mL of blood was drawn from the cephalic vein and divided into three aliquots:

Serum separator tube: for preoperative biochemical assessment, including AST, ALT, total protein, albumin, glucose, and BUN.

Cardiac stress assessment: serum NT-proBNP concentrations measured using a fluorescent immunoassay rapid test (Finecare^®^, Wondfo Biotech Co. Limited, Guangzhou, China. Samples were centrifuged at 3000 rpm for 15 min, and sera stored at −20 °C until analysis.

K3-EDTA tube: for CBC evaluation at baseline only.

### 2.7. Statistical Analysis

The minimum sample size was calculated using G*Power (version 3.1). A priori power analysis for a one-way fixed-effects ANOVA (analysis of variance) assumed an effect size (f) of 0.45, α = 0.05, power = 80%, and three experimental conditions, indicating a required total sample of 66 subjects. The sample size calculation was based on expected differences in heart rate among groups.

Statistical analyses were performed using SPSS (version 27.1; IBM Corp., Milano, Italy). Data normality was assessed via the Shapiro–Wilk test, and results are reported as mean ± standard deviation (SD). Differences across time points and between groups were analyzed using two-way repeated-measures ANOVA, followed by Bonferroni-corrected post hoc tests when applicable. Agreement between observers for postoperative pain assessment was evaluated using Kendall’s coefficient of concordance (W). SPSS automatically applied corrections when necessary. Statistical significance was set at *p* < 0.05.

## 3. Results

HR showed significant fluctuations across groups and at different time points. At baseline (T1), HR values were comparable among groups; however, during anesthesia induction (T3) and surgical stimulation (T4–T6), Group A exhibited a marked tachycardia, with significantly higher values compared to both Group C and Group O (*p* = 0.000). In contrast, Group O maintained more stable HR values, variations within ±10% of baseline values, while Group C demonstrated transient HR increases of approximately 15–20% from baseline during the surgical period (Table 1).

fR decreased significantly in Group A from T3 onwards, with values consistently lower than those observed in Groups C and O (*p* = 0.001). Group O demonstrated intermediate values, while Group C maintained higher fR across the intraoperative period (Table 2).

SAP showed an overall decline from baseline in all groups. Group O presented consistently lower SAP values compared to Groups C and A (*p* = 0.000, δ), while Group A exhibited significantly higher pressures during surgical manipulation (T3–T6) compared to the other groups (*p* = 0.001 αβδ) (Table 3).

MAP was significantly higher in Group A during anesthesia and surgery (T3–T6) compared to both Group O and Group C (*p* < 0.01). Conversely, Group O displayed a significant reduction in MAP at T2 (*p* < 0.05, *), while Group C maintained intermediate values throughout the observation period (Table 4).

DAP followed a similar trend: Group O showed significantly lower values at several time points compared to Groups A and C (*p* < 0.05), while Group A maintained higher pressures during intraoperative monitoring (*p* < 0.05) (Table 5).

Regarding NT-proBNP concentrations, no significant differences were observed at baseline (T1). However, at 48 h post-surgery (T8), Group A exhibited a marked increase in NT-proBNP levels compared to both Group C and Group O (*p* = 0.000), suggesting higher myocardial strain in this group (Table 6).

No rescue analgesia was required during either the intraoperative or postoperative period, and no adverse effects were observed.

## 4. Discussion

Maintaining cardiovascular and autonomic stability during anesthesia is critical in veterinary practice. In this context, ondansetron has emerged as a promising anesthetic adjuvant. In the present study, dogs premedicated with ondansetron maintained heart rate (HR) within physiological ranges throughout anesthesia, without episodes of clinically significant bradycardia or compensatory tachycardia. This observation underscores the rationale for investigating ondansetron’s capacity to prevent vagally mediated bradycardia while avoiding sympathetic overactivation, thereby promoting a more balanced autonomic profile.

In veterinary anesthesia, antimuscarinic agents such as atropine are routinely administered to prevent vagally mediated bradycardia [18,31]. However, the non-selective muscarinic blockade induced by atropine is recognized to cause undesirable cardiovascular effects, including pronounced tachycardia, increased myocardial oxygen consumption, reduced heart rate variability, and a potential predisposition to arrhythmogenesis [32].

Classical veterinary anesthesia texts provide foundational evidence for these pharmacological effects; Clarke, K.W. et al. (2014) describe the autonomic and chronotropic consequences of antimuscarinic administration, supporting the association between atropine use, tachycardia, and altered autonomic tone [33]. Clinical studies further substantiate the clinical relevance of these effects by characterizing cardiovascular responses to anesthetic adjuncts in small animals, with particular emphasis on increased myocardial oxygen demand and disruption of autonomic homeostasis [31]. More recent and comprehensive insights are offered by Grimm et al. (2015), who integrate advances in veterinary anesthesiology and perioperative monitoring, emphasizing the clinical implications of reduced heart rate variability and potential arrhythmogenic risk associated with antimuscarinic drugs [25].

Collectively, these sources underscore that atropine-induced cardiovascular alterations may be especially detrimental in patients with limited cardiovascular reserve or pre-existing cardiac disease. This evidence highlights the need for alternative anesthetic strategies capable of preserving autonomic balance while minimizing myocardial stress, thereby providing the rationale for evaluating ondansetron as a potential substitute. Ondansetron, a selective 5-HT_3_ receptor antagonist widely used as an antiemetic, exhibits pharmacodynamic properties that extend beyond the prevention of nausea and vomiting. By inhibiting serotonin-mediated vagal afferent signaling at both peripheral gastrointestinal sites and central autonomic nuclei—particularly the nucleus tractus solitarius—ondansetron modulates autonomic outflow and may attenuate excessive vagal activation, thereby promoting a more physiologic autonomic balance [34,35].

Ondansetron, a selective 5-HT_3_ receptor antagonist widely used as an antiemetic, exhibits pharmacodynamic properties that extend beyond the prevention of nausea and vomiting. By inhibiting serotonin-mediated vagal afferent signaling at both peripheral gastrointestinal sites and central autonomic nuclei—particularly the nucleus tractus solitarius—ondansetron modulates autonomic outflow and may attenuate excessive vagal activation, thereby promoting a more physiologic autonomic balance [34,35].

In human anesthesiology, ondansetron has gained increasing attention for its capacity to attenuate bradycardia associated with the Bezold–Jarisch reflex (BJR), a cardioinhibitory reflex mediated by stimulation of intracardiac 5-HT_3_ receptors on vagal afferent fibers and characterized by bradycardia, peripheral vasodilation, and hypotension [36]. The clinical relevance of this reflex in perioperative settings has been extensively described, particularly in association with neuraxial anesthesia and conditions of heightened vagal tone [37].

Numerous randomized clinical trials have demonstrated that prophylactic 5-HT_3_ receptor blockade with ondansetron significantly reduces the incidence and severity of Bezold–Jarisch reflex-mediated (BJR) bradycardia and hypotension in clinical settings associated with heightened vagal tone. In a randomized study of patients undergoing spinal anesthesia, preoperative administration of ondansetron effectively attenuated both hypotension and bradycardia, providing direct clinical evidence for the involvement of serotonin-sensitive vagal pathways in spinal anesthesia-induced cardiovascular instability [38]. Similarly, Charbit, B. et al. (2005), in the context of cesarean delivery under spinal anesthesia, demonstrated a significant reduction in maternal hypotension following prophylactic ondansetron administration, highlighting the clinical relevance of 5-HT_3_ receptor antagonism in obstetric anesthesia, where vagal reflexes are particularly pronounced [39]. More recently, Trabelsi, W. et al. (2015) confirmed and extended these findings in a contemporary randomized controlled trial, reporting improved hemodynamic stability during neuraxial anesthesia and reinforcing the translational applicability and reproducibility of prophylactic ondansetron use across different surgical populations [40].

The results of the present study align with these observations. Dogs receiving ondansetron-maintained heart rate values within physiological ranges throughout anesthesia, without episodes of clinically relevant bradycardia or compensatory tachycardia. In contrast, the atropine-treated group exhibited pronounced and sustained tachycardia consistent with M2 receptor blockade [41]. Importantly, ondansetron prevented vagally mediated bradycardia without provoking sympathetic overactivation, indicating a more balanced autonomic profile. Respiratory rate (fR) changes paralleled HR findings. Group A exhibited marked (fR) reductions from T3 onwards, likely reflecting autonomic disturbances induced by atropine and vagal inhibition. Conversely, ondansetron-treated dogs maintained intermediate and stable fR values, suggesting preservation of ventilatory homeostasis. These observations reinforce that ondansetron supports both chronotropic and ventilatory stability, avoiding the pronounced autonomic dysregulation observed with atropine.

Hemodynamic patterns further supported the distinct pharmacodynamic characteristics of the two agents. Atropine administration resulted in increased systolic, mean, and diastolic arterial pressures, likely reflecting enhanced sympathetic tone and elevated systemic vascular resistance. Conversely, ondansetron-treated dogs maintained lower but stable arterial pressures, with no episodes of clinically significant hypotension. These findings suggest that ondansetron avoids the exaggerated sympathetic responses associated with antimuscarinic therapy while still providing adequate vagolytic protection [41,42]. In this study, several variables (SAP, MAP, DAP) further demonstrated the differential cardiovascular impact of the two agents. Atropine administration led to significantly increased SAP, MAP, and DAP during surgical manipulation, consistent with sympathetic overactivation secondary to muscarinic blockade. In contrast, ondansetron-treated dogs maintained lower but stable arterial pressures, without episodes of clinically significant hypotension, while the control group showed intermediate values. This pattern confirms that ondansetron preserves cardiovascular stability by preventing exaggerated sympathetic responses commonly induced by atropine.

In addition, ondansetron has been associated with attenuation of surgical stress, as demonstrated in human and veterinary studies, where modulation of autonomic tone by 5-HT_3_ receptor antagonism mitigates reflex bradycardia and cardiovascular instability [17,18,19,42,43].

A particularly relevant outcome of this study concerns NT-proBNP, a validated biomarker of myocardial stretch and early cardiac stress in dogs and cats [20,23]. Indeed, NT-proBNP, as biomarker, offers a quantitative measure of organ or system functionality. It plays a key role in assessing physiological conditions, pathological states, and responses to pharmacological interventions related to cardiac distress, exhibiting high sensitivity to allow early detection, even at low concentrations typical of the initial stages of disease [44,45,46,47].

Several studies have demonstrated that NT-proBNP is particularly useful in reflecting cardiovascular stress induced by surgical or anesthetic stimuli, complementing traditional biomarkers such as cortisol and corticosterone [36,38,39]. Importantly, NT-proBNP not only indicates acute myocardial stress but also provides an integrative assessment of overall cardiac function, allowing clinicians to detect subtle changes in myocardial workload that may not be captured by traditional stress hormones alone. Several studies have demonstrated that serum and plasma concentrations of NT-proBNP in dogs and cats provide valuable support for the diagnosis and monitoring of congestive conditions and allow an indirect assessment of myocardial function. The clinical value of NT-proBNP lies in its high analytical stability, straightforward laboratory measurement, and strong sensitivity and accuracy, as well as its ability to provide insight into myocardial function. These features make NT-proBNP particularly advantageous when compared with other cardiac biomarkers that primarily reflect cardiomyocyte injury, such as troponins and creatine kinase–MB (CK-MB) [20]. NT-proBNP concentrations increased significantly at 48 h in the atropine group, consistent with sustained tachycardia, increased myocardial workload, and elevated afterload. In contrast, NT-proBNP levels remained stable in dogs treated with ondansetron, indicating that ondansetron did not impose additional perioperative cardiac stress. This observation aligns with human data showing that tachycardia and elevated myocardial oxygen demand contribute to increases in natriuretic peptides [48].

Beyond its autonomic effects, ondansetron offers clinically relevant antiemetic benefits mediated through central and peripheral serotonergic modulation [49]. Perioperative nausea and vomiting may delay recovery, increase aspiration risk, and negatively affect patient welfare; thus, the dual autonomic and antiemetic actions of ondansetron may be advantageous in multimodal anesthetic protocols, especially in procedures involving opioids or visceral manipulation.

The dual autonomic and antiemetic actions of ondansetron may be particularly advantageous in multimodal anesthetic protocols, reducing the risk of perioperative nausea and vomiting while maintaining cardiovascular stability [49].

From a clinical perspective, the combined findings of the present study reinforce the potential role of ondansetron as a preanesthetic adjuvant. Its ability to maintain stable heart rate, arterial pressure, and respiratory dynamics—together with its neutral effect on NT-proBNP—suggests that ondansetron may represent a safer alternative to atropine in patients where excessive tachycardia or increased myocardial oxygen consumption would be undesirable. This is particularly relevant for anesthetic management of animals with limited cardiovascular reserve or those undergoing procedures associated with heightened vagal stimulation.

However, ondansetron should not be considered a replacement for atropine in the management of severe, rapidly progressive, or hemodynamically compromising bradycardia. In such cases, immediate pharmacologic intervention with atropine, glycopyrrolate, or epinephrine remains essential. Instead, ondansetron may serve as a prophylactic or adjunctive agent, particularly in patients predisposed to vagal reflexes or in clinical contexts in which antimuscarinic adverse effects—such as tachyarrhythmias, increased myocardial workload, urinary retention, or exacerbation of angle-closure glaucoma—would be undesirable. Caution is warranted in patients with pre-existing QT prolongation or those receiving other QT-prolonging drugs [49]. The study’s findings also integrate well with the broader literature on the prophylactic use of ondansetron for preventing BJR-related cardiovascular instability. Although extrapolation to veterinary medicine must be cautious, this evidence strengthens the rationale for exploring ondansetron’s role in preventing reflex bradycardia in high-risk canine patients. In our study, this mechanism is supported by the finding that dogs receiving ondansetron maintained heart rate values within physiological ranges throughout anesthesia, with significantly lower heart rates than the atropine group during anesthesia induction and surgical stimulation, while also preserving stable respiratory rates and arterial pressures.

Several limitations of this study must be acknowledged. All subjects were healthy young ASA I dogs, limiting the applicability of results to animals with systemic disease, advanced age, or cardiovascular comorbidities. Only a single dosage regimen of ondansetron was examined, and no pharmacokinetic–pharmacodynamic analyses were performed. Limitations include the exclusive evaluation of a single anesthetic protocol (Met-Dia-Pro-Iso) and a single surgical procedure (elective ovariectomy). Although NT-proBNP is a robust cardiac biomarker, additional assessments—such as intraoperative echocardiography or troponin quantification—would provide a more comprehensive evaluation of myocardial stress. Lastly, the higher cost of ondansetron compared with atropine may restrict its routine use in low-risk veterinary patients. Potential perspectives for this study include investigating ondansetron’s effects in dogs with pre-existing cardiovascular comorbidities, evaluating its utility in high-vagal-tone procedures, and exploring dose–response relationships to optimize perioperative autonomic and hemodynamic stability. This contextualizes our findings and provides a rationale for future research directions in veterinary anesthesia.

## 5. Conclusions

In conclusion, ondansetron, in the dose used during anesthesia using the Met-Dia-Pro-Iso protocol, demonstrated favorable autonomic and hemodynamic stability relative to atropine, preventing bradycardia without inducing tachycardia, stabilizing arterial pressure, and avoiding perioperative increases in NT-proBNP. Combined with its antiemetic properties and potential to attenuate the BJR, ondansetron represents a promising component of multimodal anesthetic strategies aimed at reducing perioperative cardiovascular stress in canine patients.

## Figures and Tables

**Table 1:** Heart rate (HR) values at different time points. Group C (Control): animals received premedication with metadone (0.3 mg/kg) and diazepam (0.25 mg/kg). Group O: animals received premedication with ondansetron (0.5 mg/kg), metadone (0.3 mg/kg), and diazepam (0.25 mg/kg). Group A: animals received premedication with atropine (0.02 mg/kg), metadone (0.3 mg/kg), and diazepam (0.25 mg/kg).

HR (*Beats* ^*min*^)	T1	T2	T3	T4	T5	T6	T7
**Group C**	98 ± 25 αβδ	104 ± 30	129 ± 1	128 ± 4	107 ± 8	108 ± 15	103 ± 9
**Group O**	128 ± 1	136 ± 9 βδ	119 ± 2	123 ± 3	126 ± 6	124 ± 3	107 ± 10 *
**Group A**	130 ± 2	106 ± 1 *	187 ± 2 * βδ	182 ± 1 * βδ	163 ± 1 * βδ	160 ± 2 * βδ	156 ± 2 * βδ

HR = heart rate; T1 (baseline); T2 (five minutes after premedication); T3 (anesthesia); T4 (skin incision); T5 (muscle plane incision); T6 (ovariectomy); T7 (suture incisional lines). * Differences along the timeline; α differences between Group A and Group O; β differences between Group A and Group C; δ differences between Group O and Group C.

**Table 2:** Respiratory rate (fR) at different time points. Group C (Control): animals received premedication with metadone (0.3 mg/kg) and diazepam (0.25 mg/kg). Group O: animals received premedication with ondansetron (0.5 mg/kg), metadone (0.3 mg/kg), and diazepam (0.25 mg/kg). Group A: animals received premedication with atropine (0.02 mg/kg), metadone (0.3 mg/kg), and diazepam (0.25 mg/kg).

fR (*Breaths ^min^*)	T1	T2	T3	T4	T5	T6	T7
**Group C**	60 ± 2 βδ	42 ± 2 *	21 ± 2 *	20 ± 3 *	22 ± 1 *	31 ± 10 *	21 ± 3 *
**Group O**	44 ± 2	60 ± 2 * βδ	60 ± 2 * βδ	60 ± 2 * βδ	60 ± 2 * βδ	60 ± 2 * βδ	20 ± 1 *
**Group A**	32 ± 2	36 ± 3	17 ± 2	18 ± 2	18 ± 3	18 ± 3	15 ± 2

(fR) = respiratory rate; T1 (baseline); T2 (five minutes after premedication); T3 (anesthesia); T4 (skin incision); T5 (muscle plane incision); T6 (ovariectomy); T7 (suture incisional lines). * Differences along the timeline; β differences between Group A and Group C; δ differences between Group O and Group C.

**Table 3:** Systolic arterial pressure (SAP) values at different time points. Group C (Control): animals received premedication with metadone (0.3 mg/kg) and diazepam (0.25 mg/kg). Group O: animals received premedication with ondansetron (0.5 mg/kg), metadone (0.3 mg/kg), and diazepam (0.25 mg/kg). Group A: animals received premedication with atropine (0.02 mg/kg), metadone (0.3 mg/kg), and diazepam (0.25 mg/kg).

SAP	T1	T2	T3	T4	T5	T6	T7
**Group C**	140 ± 17	137 ± 14 *	109 ± 6 *	96 ± 2 *	122 ± 15 *	126 ± 8 *	123 ± 13 *
**Group O**	114 ± 10 βδ	115 ± 13 βδ	116 ± 9	117 ± 8	114 ± 10	107 ± 1	109 ± 1 βδ
**Group A**	135 ± 10	133 ± 11	136 ± 9 βδ	134 ± 7 βδ	139 ± 6 βδ	138 ± 4 βδ	127 ± 6

SAP = systolic arterial pressure; T1 (baseline); T2 (five minutes after premedication); T3 (anesthesia); T4 (skin incision); T5 (muscle plane incision); T6 (ovariectomy); T7 (suture incisional lines). * Differences along the timeline; β differences between Group A and Group C; δ differences between Group O and Group C.

**Table 4:** Mean arterial pressure (MAP) values at different time points. Group C (Control): animals received premedication with metadone (0.3 mg/kg) and diazepam (0.25 mg/kg). Group O: animals received premedication with ondansetron (0.5 mg/kg), metadone (0.3 mg/kg), and diazepam (0.25 mg/kg). Group A: animals received premedication with atropine (0.02 mg/kg), metadone (0.3 mg/kg), and diazepam (0.25 mg/kg).

MAP	T1	T2	T3	T4	T5	T6	T7
**Group C**	92 ± 13	94 ± 11	88 ± 14 βδ	90 ± 13	80 ± 3 βδ	82 ± 10 βδ	84 ± 12 βδ
**Group O**	85 ± 11	76 ± 3 βδ *	102 ± 2 *	89 ± 3	100 ± 2 *	105 ± 3 *	102 ± 2 *
**Group A**	102 ± 3 βδ	90 ± 2 *	100 ± 2	105 ± 4 βδ	103 ± 3	100 ± 2	104 ± 5

MAP = mean arterial pressure; T1 (baseline); T2 (five minutes after premedication); T3 (anesthesia); T4 (skin incision); T5 (muscle plane incision); T6 (ovariectomy); T7 (suture incisional lines). * Differences along the timeline; β differences between Group A and Group C; δ differences between Group O and Group C.

**Table 5:** Diastolic arterial pressure (DAP) at different time points. Group C (Control): animals received premedication with metadone (0.3 mg/kg) and diazepam (0.25 mg/kg). Group O: animals received premedication with ondansetron (0.5 mg/kg), metadone (0.3 mg/kg), and diazepam (0.25 mg/kg). Group A: animals received premedication with atropine (0.02 mg/kg), metadone (0.3 mg/kg), and diazepam (0.25 mg/kg).

DAP	T1	T2	T3	T4	T5	T6	T7
**Group C**	68 ± 13 δ	70 ± 14	53 ± 4 βδ *	43 ± 2 βδ *	60 ± 6 βδ	66 ± 2	61 ± 6 δ
**Group O**	75 ± 16	62 ± 4 βδ *	65 ± 14 *	66 ± 13 *	68 ± 12	53 ± 2 βδ *	57 ± 3 *
**Group A**	66 ± 2	68 ± 3	67 ± 2	68 ± 2	67 ± 2	70 ± 3	75 ± 2

DAP= Diastolic arterial pressure; T1 (baseline); T2 (five minutes after premedication); T3 (anesthesia); T4 (skin incision); T5 (muscle plane incision); T6 (ovariectomy), T7 (suture incisional lines). * Differences along the timeline; β differences between Group A and Group C; δ differences between Group O and Group C.

**Table 6:** NT-pro BNP values at different time points. Group C (Control): animals received premedication with metadone (0.3 mg/kg) and diazepam (0.25 mg/kg). Group O: animals received premedication with ondansetron (0.5 mg/kg), metadone (0.3 mg/kg), and diazepam (0.25 mg/kg). Group A: animals received premedication with atropine (0.02 mg/kg), metadone (0.3 mg/kg), and diazepam (0.25 mg/kg).

NT-proBNP (pg/mL)	T1	T8
**Group C**	176 ± 12	158 ± 31 *
**Group O**	148 ± 52	155 ± 42
**Group A**	199 ± 54	333 ± 92 * βγ

T1 (baseline); T8 (48 h after surgery). * Differences along the timeline; β differences between Group A and Group C; γ differences between Group A and Group O.

## Data Availability

The original contributions presented in this study are included in the article. Further inquiries can be directed to the corresponding authors.

## Abbreviations

The following abbreviations are used in this manuscript:

5-HT_3_	5-hydroxytryptamine type 3
SAP	Systolic Arterial Pressure
DAP	Diastolic Arterial Pressure
MAP	Mean Arterial Pressure
SpO_2_	Peripheral Capillary Oxygen Saturation
CO_2_	Carbon Dioxide
NT-proBNP	N-terminal pro–B-type natriuretic peptide
ASA	American Society of Anesthesiologists physical status
CBC	Complete Blood Count
AST	Aspartate Aminotransferase
ALT	Alanine Aminotransferase
BUN	Blood Urea Nitrogen
BJR	Bezold–Jarisch Reflex
